# Restoring cefepime activity against multidrug-resistant KPC-producing *Klebsiella pneumoniae* by combination with boronic acid inhibitors, MB076 and S02030

**DOI:** 10.1128/aac.00964-24

**Published:** 2025-01-28

**Authors:** Laura J. Rojas, Travis B. Nielsen, Paul Pantapalangkoor, Magdalena A. Taracila, Maria L. Introvigne, Rajnikant Sharma, Shekhar Yeshwante, Maria F. Mojica, Krisztina M. Papp-Wallace, Andrea M. Hujer, Fabio Prati, Emilia Caselli, Brad Spellberg, Philip N. Rather, Gauri G. Rao, Robert A. Bonomo

**Affiliations:** 1 Department of Molecular Biology and Microbiology, Case Western Reserve University School of Medicine 196211 , Cleveland, Ohio, USA; 2 CWRU-Cleveland VAMC Center for Antimicrobial Resistance and Epidemiology (Case VA CARES) 2546 , Cleveland, Ohio, USA; 3 Louis Stokes Cleveland Department of Veterans Affairs Medical Center 415208 , Cleveland, Ohio, USA; 4 Division of Infectious Diseases, Keck School of Medicine of the University of Southern California (USC) 12223 , Los Angeles, California, USA; 5 Department of Molecular Microbiology and Immunology, Keck School of Medicine of the University of Southern California (USC) 551174 , Los Angeles, California, USA; 6 Department of Medicine, Case Western Reserve University School of Medicine 220786 , Cleveland, Ohio, USA; 7 Department of Life Sciences, University of Modena and Reggio Emilia 117706 , Modena, Italy; 8 Division of Pharmacotherapy and Experimental Therapeutics, Eshelman School of Pharmacy, University of North Carolina 15521 , Chapel Hill, North Carolina, USA; 9 USC Alfred E. Mann School of Pharmacy and Pharmaceutical Sciences, University of Southern California, Los Angeles, California, USA; 10 JMI Laboratories/Element—Iowa City 138461 , North Liberty, Iowa, USA; 11 Los Angeles County-USC (LAC+USC) Medical Center 23336 , Los Angeles, California, USA; 12 Department of Microbiology and Immunology, Emory University School of Medicine 197280 , Atlanta, Georgia, USA; 13 Research Service, Atlanta Veterans Affairs Medical Center 19998 , Decatur, Georgia, USA; 14 Departments of Pharmacology, Biochemistry, and Proteomics and Bioinformatics, Case Western Reserve University School of Medicine 12304 , Cleveland, Ohio, USA; University of Fribourg, Fribourg, Switzerland

**Keywords:** BATSI, beta-lactamases, beta-lactamase inhibitor, KPC, MB076, S02030, cefepime, boronic acid inhibitor

## Abstract

Foremost in the design of new β-lactamase inhibitors (BLIs) are the boronic acid transition state inhibitors (BATSIs). Two highly potent BATSIs being developed are S02030 and MB076 strategically designed to be active against cephalosporinases and carbapenemases, especially KPC. When combined with cefepime, S02030 and MB076 demonstrated potent antimicrobial activity against laboratory and clinical strains of *Enterobacterales* expressing a variety of class A and class C β-lactamases, including *bla*_KPC-2_ and *bla*_KPC-3_. Static time-kill assays revealed the bactericidal activity of cefepime in combination with S02030 and MB076 against a multidrug-resistant KPC-producing *K. pneumoniae* (KPC-*Kpn*-1), in which a ≥ 3-log_10_ decrease in the bacterial density was observed by 6 h. *In vivo* efficacy of MB076 in combination with cefepime was evaluated in a lung infection model where male C57BL/6 mice were infected intranasally with KPC-*Kpn*-1. Cefepime alone administered at 2 h post infection resulted in a 1.07 log_10_ CFU reduction at 24 h, while cefepime in combination with MB076 resulted in an enhanced reduction of 2.70 log_10_ CFU (*P* < 0.0001) compared to the no treatment control group. In a survival analysis where mice were infected via the tail vein with KPC-*Kpn*-1*,* all mice treated with placebo or cefepime alone (100 mg/kg) died, whereas those treated with a 1:4 molar ratio of cefepime-MB076 survived. Our data demonstrate bactericidal activity and *in vivo* efficacy of cefepime-MB076 comparable to ceftazidime-avibactam and support the continued development of this combination as a new treatment option for infections caused by class A carbapenemase producing *Enterobacterales,* particularly KPC-*Kpn*.

## INTRODUCTION

Multidrug-resistant (MDR) Gram-negative bacteria pose an ongoing and significant threat to medical advancements. The emergence of these Gram-negative pathogens that are resistant to most currently approved antibiotics is concerning due to limited treatment options, which can lead to increased morbidity, mortality, and prolonged hospitalization. The World Health Organization (WHO) has identified carbapenem-resistant *Enterobacteriaceae*, *Acinetobacter baumannii*, and *Pseudomonas aeruginosa* as critical and high-priority pathogens desperately in need of new antibiotics (1). β-Lactams are the most widely used antibiotics given their wide spectrum of activity, high potency, and low adverse effect profile compared to other agents (2). Some bacteria like *Stenotrophomonas maltophilia* are intrinsically resistant to carbapenems as they have the endogenous metallo-β-lactamase (MBL) L1 that prevents the use of this class of antibiotics against these strains (3). However, in most other bacterial species, carbapenem resistance is acquired *via* horizontal gene transfer or mutational events. Hence, the emergence and rapid spread of carbapenem resistance globally is very concerning. β-Lactamase inhibitors (BLIs) in combination with susceptible β-lactams present an effective strategy to conserve the utility of these last-line agents and combat resistance. Recently, four β-lactam/β-lactamase inhibitor (BL/BLI) combinations, namely, ceftazidime/avibactam, meropenem/vaborbactam, imipenem/relebactam, and ceftolozane/tazobactam have been approved for the treatment of various carbapenem-resistant Gram-negative infections (4–7). However, evidence of the emergence of bacterial resistance to these agents is increasing, thus demonstrating the continuous need to discover new potent BLIs to retain the efficacy of β-lactam antibiotics (8, 9).

In the quest for new antibiotics, structure-based design is widely used to procure BLIs that mimic interactions observed between the target enzyme and their natural substrates (10). Attention to novel BLIs bearing an electrophilic center (phosphonates, aldehydes, trifluoromethylketones, and boronic acids) that can covalently modify the nucleophilic catalytic serine has advanced our thinking in this field (11). The boronic acid transition state inhibitors (BATSIs) have become clinically very important BLIs, as they possess a boron atom acting as an electrophile that mimics the carbonyl carbon of the β-lactam ring and can form a tetrahedral adduct with the catalytic serine that closely resembles the transition state in the hydrolytic mechanism (10, 12, 13). Presently, meropenem/vaborbactam, approved for intravenous treatment of serious nosocomial infections, attests to the interest in this class of compounds (14).

**Fig 1:**
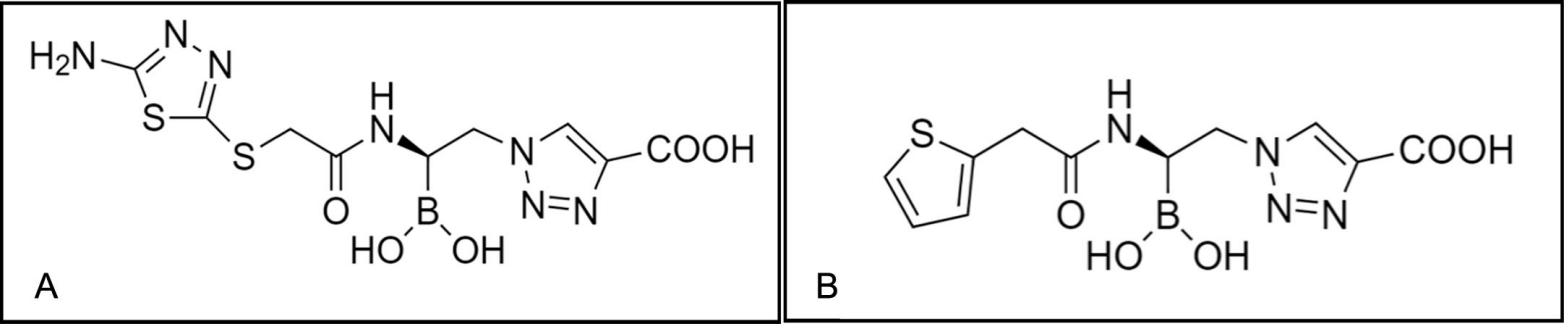
Chemical structures of (**A**) MB076 and (**B**) S02030.

Our previous studies have demonstrated the antimicrobial activity of the BATSI S02030 against *Escherichia coli* DH10B producing *bla*_SHV_, *bla*_MOX-1_, *bla*_ADC-7_, and *bla*_KPC-2 or KPC-3_ and against clinical strains of *Klebsiella pneumoniae* and *E. coli* possessing various class A β-lactamases (15, 16). Such encouraging results led to the development of novel BATSI scaffolds with a focus on improving the microbiological, pharmacokinetic (PK), and pharmacodynamic (PD) characteristics. For the design of MB076, the thiophene ring of S02030 is replaced with a 5-amino-1,3,4-thiadiazol-2-thiol ring (Fig. 1). With respect to S02030, MB076 showed a slight decrease in affinity toward KPC-2, CTX-M, and ADC variants, but an improved solubility and stability in plasma and buffer at pH 7.4 as demonstrated by *t*_1/2_ values of 29 h MB076 vs 9 h for S02030 (plasma) and *t*_1/2_ = 33 h MB076 vs 8 h for S02030 (buffer) (17).

Herein, we tested the *in vitro* activity of MB076 in combination with cefepime and evaluated the *in vivo* antibacterial efficacy of this combination in a mouse lung infection model and on the survival of mice infected intravenously with a KPC-producing *K. pneumoniae* (KPC-*Kpn*-1) isolate.

## RESULTS AND DISCUSSION

### Microbiological activity

To directly compare the microbiological activity of MB076 and its predecessor S02030 (Fig. 1), we determined the minimum inhibitory concentration (MIC) of MB076 (at a constant concentration of 4 µg/mL) in combination with cefepime by agar dilution against a panel of *E. coli* DH10B laboratory strains and 13 clinical isolates (3 *E. coli* and 10 *K*. *pneumoniae*) producing combinations of several β-lactamases, e.g., SHV, KPC, TEM, and CTX-M. Compared to cefepime alone, the addition of MB076 shifted the MIC values from resistant or susceptible-dose dependent (SDD) to susceptible, with the exception of two *K. pneumoniae* strains where the addition of MB076 changed the MIC values from the resistant to the SDD range (Table 1).

**TABLE 1:** Minimum inhibitory concentrations for *E. coli* DH10B and *E. coli* or *K. pneumoniae* clinical isolates carrying a variety of class A β-lactamases against cefepime (FEP) alone and in combination with MB076 at a fixed concentration of 4 µg/mL^*a*^

Species	Strain	β-Lactamase(s)	MIC (µg/mL)	
			FEP	FEP + MB076
*E. coli*	ATCC BAA202	SHV-1	1	≤0.25
	DH10B pBC SK control	None	≤0.25	≤0.25
	DH10B pBC SK(-) SHV-2	SHV-2	**4**	≤0.25
	DH10B pBC SK(-) KPC-2	KPC-2	**8**	≤0.25
	Eco 29838	CTX-M-14, TEM-1	**4**	≤0.25
	Eco pLTCF1	CTX-M group 9	**8**	≤0.25
	Eco pLTCF2	KPC	1	≤0.25
*K. pneumoniae*	KPC-*Kpn*-1	KPC-3, SHV-12, TEM-1	**32**	0.5
	Kpn 34700	CTX-M-15, SHV, TEM	**32**	1
	Kpn 427	TEM-1, SHV-1, CTX-M-3	**8**	≤0.25
	Kpn original KPC-2	KPC-2	**64**	≤0.25
	Kpn ST17	KPC-2, TEM, SHV, CTX-M group 1	**8**	16
	Kpn ST258 C3	KPC-3	**32**	1
	Kpn ST258 B5	KPC-2	**32**	8
	Kpn 253	TEM-12, SHV-5, CTX-M	**32**	8
	Kpn266	SHV-5	2	≤0.25
	Kpn 255	TEM-1, SHV-2	**4**	2

^*a*^ MICs in bold indicate values that are susceptible dose-dependent (SDD) or resistant according to CLSI guidelines (18).

**Fig 2:**
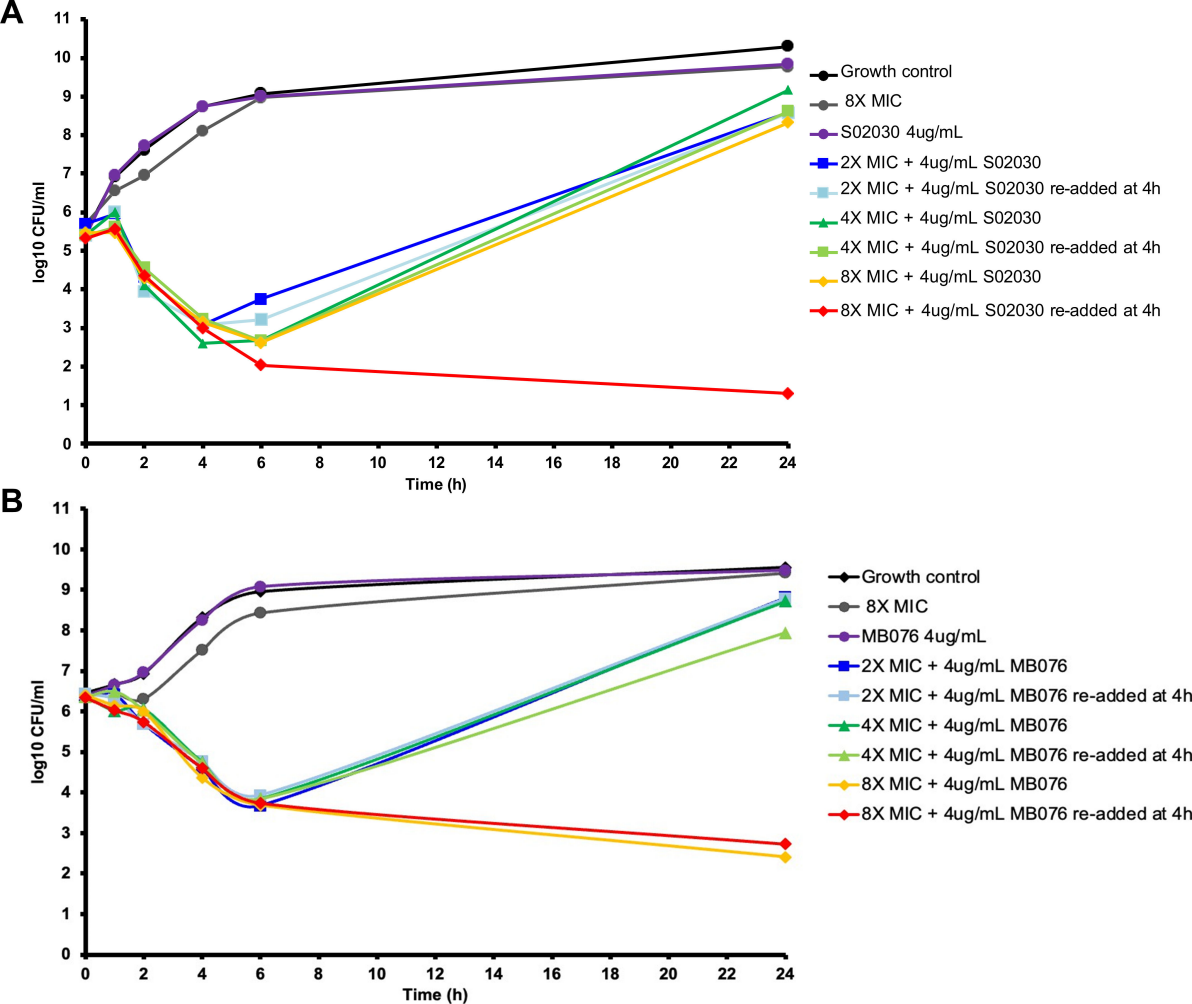
Static time-kill assays for cefepime alone and in combination with S02030 (**A**) or MB076 (**B**). Mean bacterial concentrations over 24 h against an initial inoculum of 10^6^ CFU/mL of *K. pneumoniae* KPC-*Kpn*-1 were calculated using cefepime concentrations corresponding to 2×, 1 µg/mL; 4×, 2 µg/mL; and 8×, 4 µg/mL the MIC of the combination cefepime-S02030/cefepime-MB076 by agar dilution (0.5 µg/mL). A growth control (no antibiotics added) and cefepime alone at 8× the combination MIC were included. Inhibitor at 4 µg/mL was added at 4 h (indicated by re-added at 4 h). Three replicates were conducted for each of the conditions reported.

The clinical isolate KPC-*Kpn*-1 recovered from a bloodstream infection, displayed an MIC of 32 µg/mL for cefepime alone and 0.5 µg/mL cefepime in combination with a fixed concentration of 4 µg/mL of either S02030 or MB076, a sixfold reduction in MIC. Based on these results, the effect over time of this combination was evaluated by performing *in vitro* time-kill assays over 24 h. A range of cefepime concentrations corresponding to 2×, 4×, and 8× the MIC (0.5 µg/mL) of cefepime in combination with S02030 resulted in a 3-log reduction in bacterial density by 4 h for all conditions evaluated, followed by regrowth similar to control growth by 24 h. The impact of degradation of the inhibitor at 37°C was assessed by supplementing with 4 µg/mL S02030 at 4 h, resulting in sustained bactericidal activity beyond 6 h differentiating it from the other tested conditions. (Fig. 2A). Similarly, cefepime in combination with MB076 resulted in ~3 log reduction in bacterial density by 6 h. Cefepime 8× MIC in combination with MB076 displayed sustained bactericidal activity beyond 6 h unlike cefepime 8× in combination with S02030, which resulted in regrowth similar to the control by 24 h. MB076 demonstrated sustained bacterial killing at 4 µg/mL, while S02030 achieved sustained killing at 8 µg/mL, indicating that MB076 is a more effective inhibitor (Fig. 2B).

**Fig 3:**
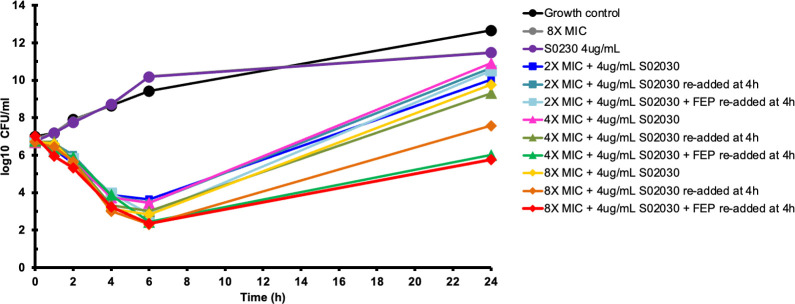
Static time-kill assays in 50% human serum. Mean bacterial concentrations over 24 h against an initial inoculum of 10^6^ CFU/mL of *K. pneumoniae* KPC-*Kpn*-1 were calculated using cefepime concentrations corresponding to 2×, 1 µg/mL; 4×, 2 µg/mL; and 8×, 4 µg/mL the MIC of the combination cefepime-S02030 by agar dilution (0.5 µg/mL). A growth control (no antibiotics added) and cefepime alone at 8× the combination MIC were included. Inhibitor at 4 µg/mL (indicated by re-added at 4 h) or full initial dose of cefepime and S02030 (indicated by FEP+ S02030 re-added at 4 h) was added at 4 h. Three replicates were conducted for each of the conditions reported.

The effect of protein binding on the microbiological activity of cefepime + S02030 was evaluated by performing time-kill assays in 50% human serum. The results are comparable to those obtained in Muller Hinton broth (MHB) alone, with a bacterial density decrease of 3-log by 6 h for most of the conditions evaluated (Fig. 3). However, in contrast with the previous results, regrowth was observed in all conditions, even when cefepime and S02030 were replenished.

Our *in vitro* results demonstrate that S02030 and MB076 restore cefepime activity against a multidrug drug-resistant KPC-*Kpn* clinical isolate. Regrowth has been previously observed with other BL-BLI combinations, including the recently developed ceftazidime/avibactam (19). Cefepime-S02030 resulted in bactericidal activity by 6 h both in the absence and presence of human serum (Fig. 2A and 3).

### Pharmacokinetics of MB076 following single IV dose in infected mice

**Fig 4:**
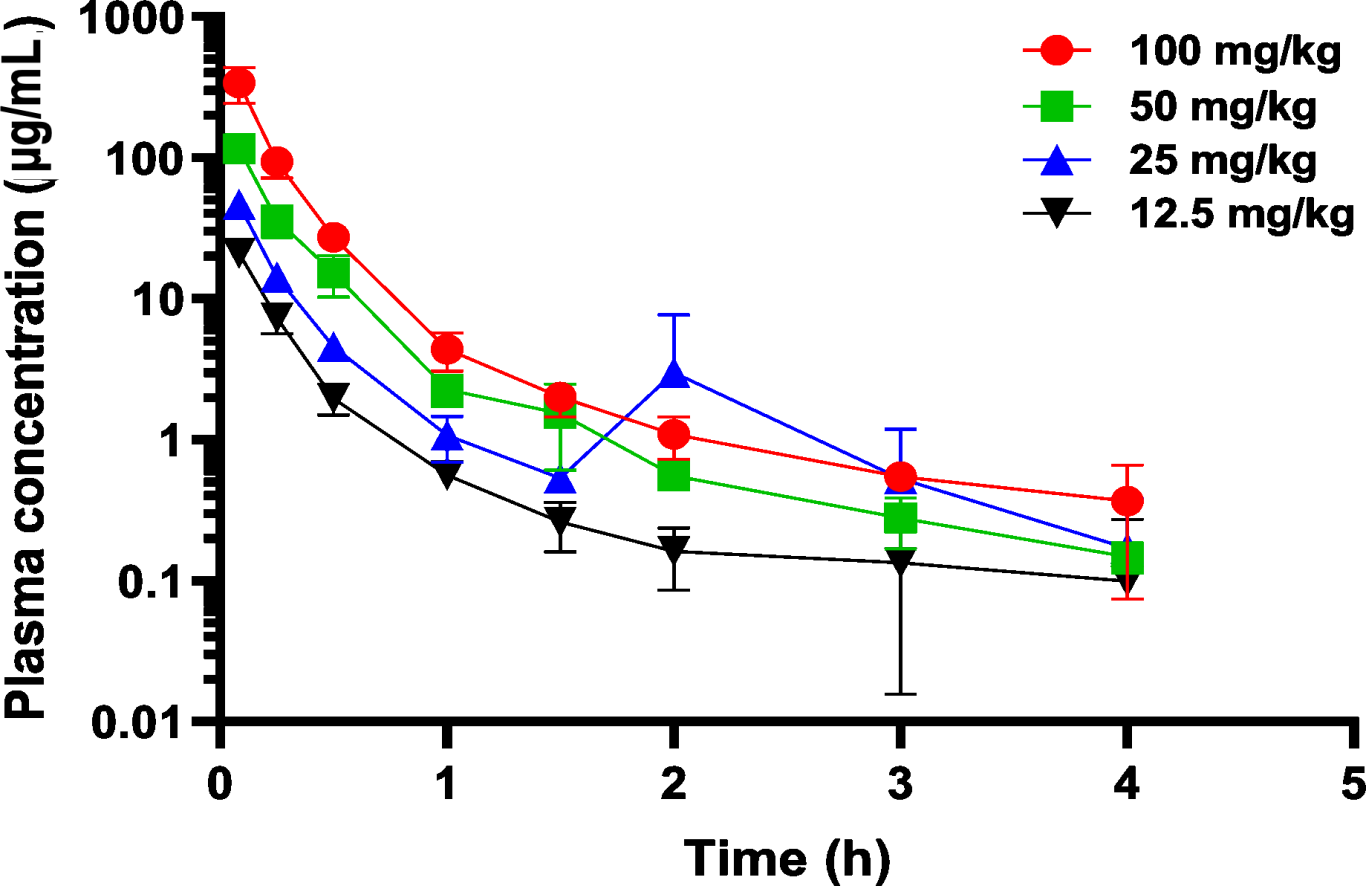
The mean plasma concentration vs time profiles of MB076 after IV administration of a single MB076 dose to thigh infected mice. Error bars are SD values, with a limit of detection of 0.1 µg/mL.

A dose-ranging PK study was conducted to evaluate the PK of MB076 following the administration of a single IV dose to mice infected with KPC-*Kpn*-1. The range of MB076 doses evaluated included 12.5, 25, 50, and 100 mg/kg (Fig. 4).

The AUC_last_ calculated for each of the dose levels ranged from 7.1 to 103.2 µg/mL, and it increased in a dose proportional manner indicating linear PK. The peak plasma concentration at time = 0 h, *C*_0_ ranged from 35.6 to 638 µg/mL (Table 2).

**TABLE 2:** Plasma pharmacokinetic parameters for MB076 after single IV administration to thigh-infected mice

Dose, IV (mg/kg)	*t*_1/2_ (h)	C0 (µg/mL)	AUC_last_ (µg·h/mL)	AUC_inf_ (µg·h/mL)	Vss (L/kg)	CL (mL/min/kg)
12.5	0.56	35.6	7.1	7.2	0.65	29.1
25	0.61	84	17.6	17.8	0.80	23.4
50	0.43	207	39	31.9	0.35	21.3
100	0.43	638	103	104	0.21	16.1

MB076 is a low clearance compound with an extraction ratio of 0.33 (hepatic blood flow in mouse 5.4 L/h/kg). The volume of distribution is higher than plasma volume and has a half-life of 2.85 h (12.5 mg/kg). MB076 shows linear PK in plasma up to 50 mg/kg dose. At 100 mg/kg, MB076 shows non-linear behavior with a more than dose-proportional increase in AUC and 1.7-fold reduction in clearance compared to a lower dose of 12.5 mg/kg. The dose-normalized AUC increases by twofold from 12.5 to 100 mg/kg dose (Fig. 4).

### *In vivo* efficacy of cefepime and MB076 combination regimens

**Fig 5:**
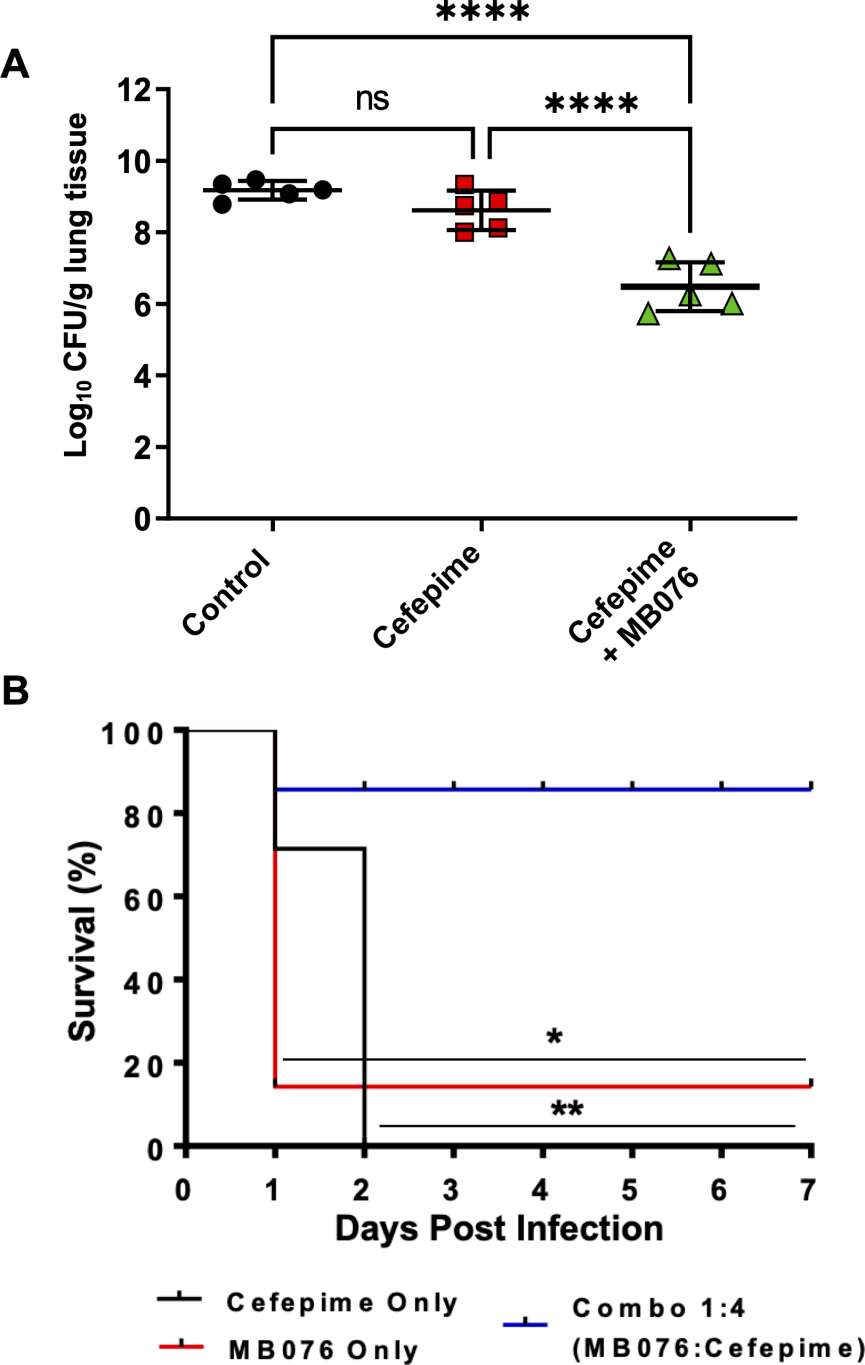
(**A**) Bacterial burden 24 h post initiation of treatment in BALB/c mice (*n* = 5) infected intranasally with *Klebsiella pneumoniae* KPC-*Kpn*-1. Treatment was initiated 2 h post infection: group 1: control no treatment; group 2: cefepime alone; and group 3: cefepime in combination with MB076. Cefepime was dosed 127.5 mg/kg/day subcutaneously, and MB076 was dosed 100 mg/kg/day intravenously. Statistical analysis was performed by a one-way ANOVA followed by Tukey’s multiple comparison test, ****P < 0.0001 significant, *P < 0.05, ns, nonsignificant. (**B**) Kaplan-Meier survival curves were estimated over 7 days. Seven male C57BL/6 mice per treatment were infected IV via the tail-vein with 2.1 × 10^8^ CFU/mouse with *K. pneumoniae* KPC-*Kpn*-1 and treated with 100 mg/kg/dose of cefepime, MB076, cefepime + MB076 (1:4 ratio), or placebo at the time of infection. *P < 0.05, **P < 0.01 significant.

The bacterial load in the lungs of neutropenic mice induced by intranasal challenge with *K. pneumoniae* isolate KPC-*Kpn*-1 was assessed at 0 and 26 h post infection. At 0 h, the average bacterial burden was 7.07 ± 0.14 log_10_ CFU/g lung, and this increased to ~9 log_10_ CFU/g lung in untreated controls over 26 h. In mice infected with KPC-producing isolate KPC-*Kpn*-1, cefepime alone administered at 2 h post infection resulted in a 1.07 log_10_ CFU reduction at 24 h compared to the no-treatment control group. While cefepime in combination with MB076 resulted in a significant reduction of 2.70 log_10_ CFU (*P* < 0.0001) compared to the no-treatment control group at 24 h post treatment (Fig. 5A).

### Survival analysis

We tested the efficacy of cefepime with or without MB076 on the survival of mice infected intravenously with KPC-*Kpn*-1. The infected animals were treated with placebo (control), cefepime, or cefepime + MB076 (at 1:1 or 1:4 ratio). Doses were repeated the following morning and afternoon (at 18 and 24 h post infection, respectively). All mice treated with cefepime alone died, whereas all mice treated with combination therapy at a 1:4 molar ratio survived. (Fig. 5B).

In conclusion, we have demonstrated *in vitro* bactericidal activity of cefepime in combination with two BATSIs: S02030 and the novel MB076. The *in vivo* efficacy of MB076 in combination with cefepime demonstrated ~3 log reduction of KPC-*Kpn*-1, demonstrating excellent penetration in the lung. This combination also had an impact on survival compared to monotherapy with cefepime alone. These results support the continued development of this combination as a new treatment option for treating infections caused by class A carbapenemase-producing *Enterobacterales*, particularly KPC-*Kpn*.

## MATERIALS AND METHODS

### Antimicrobials and media

Cation adjusted (25.0 mg/L Ca^2+^ and 12.5 mg/L Mg^2+^) Mueller-Hinton broth (CAMHB; BBL Microbiology Systems, Cockeysville, MD) and agar (CAMHA; Becton, Dickinson and Company, Sparks, MD) were used for susceptibility testing and for all *in vitro* experiments. Stock solutions of cefepime (lot number WXBB5309V; Sigma-Aldrich, St. Louis, MO), S02030, and MB076 were freshly prepared in sterile water prior to each experiment. All antibiotic solutions were filter sterilized using a 0.22-μm-pore-size filter (Fisher Scientific, Pittsburgh, PA).

### Minimum inhibitory concentration determination

MICs for cefepime alone and in combination with a fixed concentration of 4 µg/mL of MB076 (Fig. 1) were determined in triplicate by agar dilution as per Clinical and Laboratory Standards Institute (CLSI) guidelines (18). The combination of cefepime and MB076 was tested against a wide panel of laboratory and clinical strains producing different class A and C β-lactamases (Table 1), including KPC-*Kpn*-1, a clinical KPC-*Kpn* multidrug-drug-resistant bloodstream isolate used in subsequent *in vitro* and *in vivo* assays.

### Static time-kill assays

Static time-kill assays were performed for KPC-*Kpn*-1, a clinical KPC-harboring *K. pneumoniae* multidrug-resistant bloodstream isolate as previously described (19). Briefly, freshly prepared colonies grown overnight on blood agar were resuspended in 5 mL CAMHB and incubated in a shaking incubator (37°C, 220 rpm) until they reached the 0.5 McFarland standard (approximately 10^8^ CFU/mL). A bacterial suspension was prepared in 50 mL conical tubes containing 10 mL CAMHB to obtain a starting inoculum of approximately 10^6^ CFU/mL. For assays testing the effect of serum, bacterial suspensions were diluted in 100% human serum so that the final concentration of human serum in CAMH was 50% (19). Cefepime was added to the prepared bacterial suspensions so that the final concentration was 1×, 2×, 4×, or 8× the agar MIC for the cefepime + MB076 or cefepime + S02030 combination, while the inhibitor was held constant at 4 µg/mL. Controls consisting of cefepime alone diluted in the prepared bacterial suspension at 8× the MIC and a growth control without antibiotic were also included. The starting inoculum was determined from the growth control tube immediately after dilution and was recorded as the count at 0 h. Tubes were incubated in a shaking incubator (37°C, 180 rpm), and samples were obtained at 1, 2, 4, 6, and 24 h for bacterial quantification by plating 100 µL of the appropriately diluted sample following serial 10-fold dilutions in sterile CAMH broth on CAMH agar plates. Plates were incubated at 37°C for 18–20 h, and bacterial colonies were counted with a limit of quantification of 20 CFU/mL. Three replicates were conducted for each of the conditions evaluated in the time-kill assay. The results were analyzed by calculating the reductions in log_10_ CFU/mL at each time point, compared to bacterial counts at time *t* = 0 h for each of the antibiotic concentrations evaluated.

### Bacterial inoculum preparation for *in vivo* studies

A 0.2 mL aliquot of a single-use glycerol stock of the KPC-*Kpn* isolate (at −80°C) was used to seed 20 mL brain heart infusion (BHI) and then incubated at 35–37°C with shaking (120 rpm) for 8 h. Bacterial cells in the 20 mL aliquot were pelleted by centrifugation (3,500 × *g*) for 15 min and then resuspended in 10 mL cold phosphate buffer saline (PBS). The optical density, OD_620_ nm, was measured and used to guide dilution. The PBS suspensions were stored on ice for no more than 2 h prior to infecting the animals. The bacterial count in the challenge organism suspension was enumerated by dilution plating to CAMH agar plates followed by 20–24 h incubation.

### *In vivo* lung infection model

Eleven- to 12-week-old BALB/c mice obtained from Charles River Laboratories (Raleigh, NC) were allowed to acclimatize for at least 1 week prior to initiating the studies. All animal procedures were approved by the Institutional Animal Care and Use Committee at the University of North Carolina at Chapel Hill (IACUC ID: 21-125.0), and all experiments were performed in accordance with institutional guidelines for animal experiments.

### Induction of neutropenia

Cyclophosphamide (Sandoz Inc, Princeton, NJ; Lot no #22041725) was dissolved in normal saline for injection to a final concentration of 25 mg/mL stock solution. Mice were rendered neutropenic with the administration of two intraperitoneal injections on day −4 (75 mg/kg single dose) and day −1 (50 mg/kg single dose) prior to bacterial inoculation.

### Intranasal inoculation of infection

Mice were anesthetized by inhalation of 2% isoflurane mixed with oxygen using an isoflurane vaporizer chamber. Mice were held in a vertical position at a 45° incline to ensure that the bacteria reached the lower parts of the lung. Bacterial suspension of 20 µL was instilled into each nostril as 4 drops with 5 µL per drop using a micropipette. Animals were maintained in the vertical position until they recovered from anesthesia (20).

### Antibiotic treatment

Antibiotic treatment was initiated 2 h post infection. Mice were randomized to control or treatment groups [group 1: control (no treatment), group 2: cefepime only, and group 3: cefepime in combination with MB076]. Animals in the control group were treated with saline. Mice in group 2 received a cefepime dose of 127.5 mg/kg/day subcutaneously (WG critical Care LLC, Paramus, NJ; Lot no #109804C). Mice in Group 3 received cefepime 127.5 mg/kg/day subcutaneously in combination with MB076 dose of 100 mg/kg/day (25 mg/kg every 6 h) administered via intravenous route. The total daily cefepime dose of 127.5 mg/kg/day was administered as 30, 10, and 2.5 mg/kg of cefepime at 0, 3, and 6 h every 8 h. The dosing schedule ensures a *f*T > MIC of >60% for bacteria with MIC of ≤16 µg/mL.

The cefepime dose was selected based on PK/PD index for cefepime and human exposure achieved at clinical doses. The PK/PD index that best describes the relationship between cefepime PK and PD is the percentage of the dosing interval during which the unbound drug concentrations exceed the MIC (%*fT* > MIC) in human plasma. Craig et al. reported that %*fT* > MIC of 50%–70% is necessary for cephalosporin bactericidal activity (21, 22). Literature reports suggest the use of breakpoint for the estimation of target attainment for cefepime (23–25). The latest CLSI breakpoints for cefepime against Enterobacteriaceae are ≤2 (S), 4–8 (I), and ≥16 (R) (18). We evaluated the %*fT* exceeded MIC of ≤16 µg/mL during the 8 h dosing interval in mouse. Our simulated cefepime dosing regimen of 30, 10, and 2.5 mg/kg dosed at 0, 3, and 6 h during each 8 h interval provides a *f*T > MIC of >60% for bacteria with MIC of ≤16 µg/mL. The selected dosing regimen simulated in our mouse studies was based on the free drug exposure of 189–308 mg·h/L achieved at the currently approved cefepime dose of 2 g every 8 h taking the plasma protein binding of 16%–19% observed in healthy subjects into consideration (26, 27). The simulated free exposure achieved taking plasma protein binding in mice of 0%–5% into consideration was within the range of drug exposure achieved in humans (211 mg·h/L) (28, 29). A total cefepime dose of 127.5 mg/kg/day was injected in mice over 24 h to achieve the humanized cefepime drug exposure.

The second consideration regarding the cefepime dosing regimen for the *in vivo* mouse studies was the partitioning of the drug penetration into the lung and epithelial lining fluid (ELF) to ensure adequate drug exposure required for effective killing activity. Johnson et al. evaluated the *in vivo* activity of cefepime and enmetazobactam in neutropenic animals against three ESBL-producing strains (30). The penetration into the lung was determined by comparing the area under the concentration-time curve (AUC) in plasma and epithelial lining fluid (AUC_plasma_/AUC_ELF_). The AUC_ELF_:AUC_plasma_ ratio for cefepime was 73.4%. Based on a phase 1 multidose open label study conducted in healthy adults to evaluate the PK of cefepime and zidebactam, Rodvold et al. reported median AUC_ELF_/AUC_plasma_ of 38%. They reported an increased penetration to ELF over time (ratio of ELF to total plasma concentration ratio of 0.31 ± 0.07 at 0.5 h that increased to 0.74 ± 0.42 at 10 h) (31). We anticipate that our proposed regimen will result in adequate penetration of cefepime to ELF and lung tissue to be efficacious with MB076.

### Quantification of bacterial load in lung samples

Five mice/group were sacrificed by overdose of inhaled 5% isoflurane at 26 h post inoculation. Lungs were removed aseptically, weighed, and homogenized in ~2 mL of PBS (Source of PBS) using Polytron PT2100 homogenizer (Kinematica, Inc, NY). Homogenates were serially diluted 10-fold in PBS and plated on MHA plates. The plates were incubated at 37°C for 24 h, after which the bacterial density per gram of lung was enumerated. Lung bacterial counts were analyzed using a one-way ANOVA followed by *post-hoc* Tukey multiple comparison test (GraphPad Prism 10.1, GraphPad Software Inc., San Diego, USA). A *P* value of < 0.05 was considered statistically significant.

### Survival studies

Seven male C57BL/6 mice per treatment group were infected intravenously via the tail-vein by injecting a prepared inoculum of KPC-*Kpn*-1, input CFU = 2.1 × 10^8^ CFU/mouse, contained in a volume of 0.25 mL per animal. Animals were treated with intraperitoneal 100 mg/kg/dose of cefepime, MB076, cefepime + MB076 (1:4 ratio) or placebo at the time of infection. Doses were repeated the following morning and afternoon (18 and 24 h after infection). Survival was evaluated 24 h post-treatment. Kaplan-Meier survival curves were generated using log-rank test (GraphPad Prism 10.1, GraphPad Software Inc., San Diego, USA). A *P* value of <0.05 was considered statistically significant.

### Evaluation of MB076 pharmacokinetics in infected mice

A single dose PK study was conducted in mice infected with *K. pneumoniae* AR-BANK#0098 (producing KPC-2, OXA-9, and TEM-1) employing a thigh infection model. Escalating doses of MB076 (12.5, 25, 50, and 100 mg/kg) were intravenously administered via the tail vein. Following this terminal blood sampling via cardiac puncture was performed at eight different time points 5, 15, 30, 60, 90, 120, 180, and 240 min for each dose level. Plasma was separated by centrifugation at 4,000*g* for 10 min at 4°C and stored at −80°C until MB076 quantification was performed by LC/MS-MS bioanalysis.

WinNonlin software (Phoenix Build 8.3.1.5014) was employed to perform noncompartmental analysis (NCA) to calculate the PK parameters like elimination half-life (*t*_1/2_), area under the concentration–time curve over the duration of study (AUC_0–last_), and infinity (AUC_0–inf_), and the peak drug concentration (C_0_), volume of distribution (V) and clearance (CL). The AUCs for the different MB076 dose levels were calculated and compared to assess for any dose related non-linearity with increasing dose.

### Plasma collection and processing for LC-MS/MS analysis

Terminal blood collection from cardiac puncture was conducted under CO_2_ euthanasia. Blood, 0.3–0.4 mL, was drawn into tubes coated with K2EDTA, mixed gently and kept on ice, and centrifuged at 12,000 × *g* for 5 min at 4°C within 1 h of collection. The plasma was harvested and stored at −80°C until further processing.

A 20 µL aliquot of each plasma sample was transferred to a 96 well plate. A 500 µL aliquot of 0.01 ng/µL internal standard (IS), Oxybutynin, in ACN/FA = 95/5, was added to each tube except for the double blank and carryover controls. A 500 µL aliquot of ACN/FA = 95/5, without oxybutynin, was added to the double blank and carryover assay wells. The equilibrated mixture was vortexed for 1 min and then centrifuged at 4,000 rpm for 10 min at 20°C to precipitate protein. After centrifugation, 500 µL of the supernatant was transferred to a new well of the 96 well plate for LC-MS/MS analysis. Plasma samples from treatment groups and the calibration curve samples were processed on the same test occasion using the same procedure. The calibration curve samples were generated by spiking aliquots of drug-free plasma with an MB076 dilution. The quality control (QC) samples, QH (high), QM (medium), and QL (low) were each assayed in duplicate.

### LC-MS/MS analysis

LC-MS/MS was conducted with a SCIEX Triple Quad 5500 + mass spectrometer. Method development was conducted to determine appropriate conditions for LC-MS/MS analysis of MB076. A titration of a stock solution was performed to correlate peak areas and the corresponding concentration. The reportable linear range of the assay was determined along with the lower and upper limits of quantitation (LLOQ and µOQ).

To develop the bioanalytical method, MB076 was prepared in ACN/H2O = 50/50 at 1 ng/µL. Full scan MS analyses were conducted, and total ion current chromatograms and corresponding mass spectra were reviewed for MB076 in both positive and negative ionization modes. The precursor ions for MS/MS were selected from either the positive or the negative mass spectrum, as a function of the respective ion abundance. In addition, product ion MS/MS analysis was performed to determine the appropriately selected fragmentation reaction for use in quantitative analysis. The final reaction monitoring parameters were chosen to maximize the ability to quantify the test compound when present within a complex mixture of components. Following the identification of the specific multiple reaction monitoring (MRM) transition to be used for each test compound, the detection parameters were optimized. MB076 was observed as one charged ion [M + H]+ and the MRM chosen was *m*/*z* = 374.0 to *m*/*z* = 243.2 for MB076 in electrospray positive ion mode.

The chromatographic conditions used for LC-MS analysis were identified by injection and separation of the analyte on a suitable LC column followed by adjustment of the gradient conditions as necessary. In brief, the chromatography was done with the XSelect Peptide HSS T3 XP column 2.5 µm (2.1 × 150 mm) column. LC was conducted with an isocratic elution of H2O/FA = 99/1 (vol/vol) (mobile Phase A) and ACN/MeOH/FA = 80/20/1 (vol/vol) (mobile Phase B). The total run time was 4 min, and the injection volume was 2 µL.
